# Peanut immunotherapy

**DOI:** 10.1186/2045-7022-4-30

**Published:** 2014-09-25

**Authors:** Katherine Anagnostou, Andrew Clark

**Affiliations:** 1 Department of Paediatric Allergy, Guy’s and St Thomas’ Hospitals NHS Foundation Trust, Westminster Bridge Road, London, UK; 2 Department of Medicine, University of Cambridge, Addenbrooke’s Hospital, Box 157, Cambridge CB2 0QQ, UK

**Keywords:** Allergy, Peanut, Immunotherapy

## Abstract

Peanut allergy is common and can be a cause of severe, life-threatening reactions. It is rarely outgrown like other food allergies, such as egg and milk. Peanut allergy has a significant effect on the quality of life of sufferers and their families, due to dietary and social restrictions, but mainly stemming from fear of accidental peanut ingestion. The current management consists of strict avoidance, education and provision of emergency medication, but a disease- modifying therapy is needed for peanut allergy. Recent developments involve the use of immunotherapy, which has shown promise as an active form of treatment. Various routes of administration are being investigated, including subcutaneous, oral, sublingual and epicutaneous routes. Other forms of treatment, such as the use of vaccines and anti-IgE molecules, are also under investigation. So far, results from immunotherapy studies have shown good efficacy in achieving desensitisation to peanut with a good safety profile. However, the issue of long-term tolerance has not been fully addressed yet and larger, phase III studies are required to further investigate safety and efficacy. An assessment of cost/benefit ratio is also required prior to implementing this form of treatment. The use of immunotherapy for peanut allergy is not currently recommended for routine clinical use and should not be attempted outside specialist allergy units.

## Introduction

Peanut allergy was once rare, but is now the most common cause of fatal food-allergic reactions [1]. The prevalence has increased steadily over the past decade, mostly in the Western World, the disease currently affecting 1-2% of children [2-4]. In two different case series of fatal food-allergic reactions published in 1992 and 2001 respectively, peanut was a common cause [1,5]. Accidental reactions are common, as peanuts can be hidden in various foods or contaminate meals in restaurants [6,7]. Peanut allergic individuals experience lower quality of life due to high levels of anxiety and increased awareness that their condition can be fatal; they also feel that they have less control over their disease compared with diabetic children [8]. Parents of peanut allergic children also present high levels of stress, mainly due to their child’s risk of death and constant dietary restrictions [9].

In contrast to other food allergies, such as egg and milk - both of which are usually outgrown in the majority of patients - only a small percentage of children are expected to outgrow their peanut allergy (approximately 20% based on published studies) [10-13]. There is therefore a clear need for a disease-modifying treatment.

Experience drawn from immunotherapy trials in allergic rhinitis and venom allergy is positive. Subcutaneous immunotherapy (SCIT) for pollen-induced rhinitis is a successful therapy which is disease-modifying, in that it results in long-lived tolerance in most individuals after a three year course [14,15]. Sublingual immunotherapy (SLIT) has been shown to significantly reduce both rhinitis symptoms and the requirement for anti-allergic medication [16]. Further success is seen in SCIT insect venom immunotherapy, where it is possible to safely desensitise patients with life-threatening reactions [17]. In children, venom immunotherapy has shown high efficacy in preventing systemic reactions after subsequent insect stings [18-20].

Studies on food allergy immunotherapy for milk and egg have shown promising efficacy in desensitising allergic children. Longo et al. designed a large RCT on milk immunotherapy, involving 60 children, all with previous severe reactions to milk. After 12 months of OIT, 36% of participants became completely tolerant to 150 mls of cow’s milk, whereas 54% became partially tolerant and 10% failed [21]. A recent systematic review on milk immunotherapy has reported that oral immunotherapy, when compared to an elimination diet alone, increased the likelihood of achieving full tolerance to cow’s milk [22]. Egg oral immunotherapy has also been successful in achieving desensitisation in patients with egg allergy [23]. However, both milk and egg allergies tend to self-resolve, so it can be difficult to assess the effect of immunotherapy versus natural allergy resolution.

## Review

### Subcutaneous immunotherapy

Subcutaneous peanut immunotherapy was attempted in a small study in 1992, where an initial rush injection schedule was administered, followed by once-weekly maintenance injections for 4 weeks. Three subjects in the active group had a 67-100% reduction in symptoms induced by peanut challenge, suggesting this is an effective form of therapy. The rate of systemic reactions was high (13.3%). Unfortunately, the study was terminated early due to a fatal reaction, following a formulation error in the pharmacy, which resulted in the accidental administration of a maintenance dose of peanut to a placebo-treated subject. The subject died of anaphylaxis. It was concluded that subcutaneous immunotherapy to peanut showed potential as a form of therapy, but the safety profile of such an intervention required further study [24] (Additional file 1).

A subsequent study investigated the effect of injections of peanut extract in achieving desensitisation to peanut in 12 adult patients (split equally between active and control group), with immediate hypersensitivity following peanut ingestion. Although the administration of subcutaneous immunotherapy resulted in an increase in peanut threshold for the subjects in the active group, assessed by a DBPCFC, it was associated with repeated systemic reactions, even during maintenance injections (23% of subjects during rush immunotherapy and 39% during maintenance experienced systemic reactions) [25]. This high rate of systemic reactions made this form of treatment unacceptable for routine use in peanut allergic subjects and different routes of administration of peanut allergen were subsequently examined as discussed below.

### Oral immunotherapy (OIT)

An open pilot study of peanut oral immunotherapy undertaken in the UK in 22 peanut-allergic children, reported that 86% of participants tolerated 5 peanuts daily after a median of 140 days of oral immunotherapy and were all protected from amounts likely to be ingested accidently. There was a median 1000-fold increase in the amount of peanut tolerated by subjects (from 6 mg at baseline to 6,459 mg post immunotherapy) following intervention, with a good safety profile. Reactions were mostly mild and no adrenaline was administered during oral immunotherapy. A novel protocol with gradual up dosing and high maintenance dose was used, resulting in a better safety profile and outcome [26,27].

A US open study of oral peanut immunotherapy reported on 29 subjects, 1–9 years of age with peanut allergy, who underwent a protocol of initial day escalation, build-up and maintenance phase. The majority (93%) of participants who completed the OIT protocol were successfully desensitised and tolerated a final challenge of 3.9 g of peanut protein. Most symptoms observed during the intervention resolved spontaneously or with the use of antihistamines, but 4 subjects required adrenaline administration during the rush phase and 2 subjects during the build up and maintenance phases [28].

In the same year, a randomised controlled study of oral peanut immunotherapy included 19 children who completed a year of OIT (initial escalation phase, home dosing, build up visits and maintenance phase). The investigators reported that 84% of subjects passed a final challenge of 20 peanuts, successfully ingesting 5 g of peanut protein compared with only 1 peanut or 280 mg of peanut protein (median value) ingested by the placebo subjects. The authors concluded that the degree of protection following successful immunotherapy was likely to prevent accidental peanut anaphylaxis [29].

The largest phase II, randomised-controlled, crossover trial of peanut oral immunotherapy (OIT) was recently published in the Lancet, investigating the role of peanut oral immunotherapy in desensitising 99 children inclusive of all severities of peanut allergy. In the active group, 84% were desensitised to 800 mg (approximately 5 peanuts), whereas 24 of 39 (62%) OIT participants were successfully desensitised to 1,400 mg of peanut protein (approximately 10 peanuts). Subjects who successfully completed the study protocol had a significant 25-fold increase of their peanut threshold, and their caregivers had a significant improvement in quality of life. Adverse effects seen in most participants, were mild and easily treatable. Adrenaline was administered to one subject with prompt resolution of symptoms [30].

It is clear from the above studies that peanut oral immunotherapy presents an interesting novel form of intervention for peanut-allergic children, resulting in good efficacy for desensitisation. The safety profile is also good with most subjects experiencing mild or moderate reactions.

### Sublingual immunotherapy (SLIT)

A study of sublingual peanut immunotherapy was published in 2011. In this double blind placebo controlled study, all 18 participants underwent a 6-month period of dose escalation, followed by a 6-month period of maintenance therapy. Side effects consisted of mostly oropharyngeal symptoms and only 0.3% of doses required antihistamine treatment. A DBPCFC was used to assess the final outcome, following a year of treatment, which showed the treatment group safely ingesting 20 times more peanut protein than the placebo group (1,710 mg versus 85 mg) [31].

A subsequent multi-centre, randomised, placebo-controlled trial of peanut SLIT has shown a modest effect in desensitisation to peanut. After 44 weeks of treatment, clinical desensitisation was observed in 70% of the active and 15% of the placebo subjects. The median successfully consumed dose for the active group increased form 3.5 mg at baseline to 496 mg peanut flour (approximately 50% peanut protein) after a year of therapy, but none of the participants were able to pass a 5 g peanut challenge (the study’s primary outcome). The safety profile was very favourable with 59.9% of doses in the active group being symptom-free, and once oropharyngeal symptoms were excluded, the percentage rose to 94.7% of symptom-free doses [32].

Although SLIT appears to have a very good safety profile, the effect of desensitisation is modest compared with OIT. The allergen doses are much lower in SLIT due to practical limitations and this limits its efficacy. More research studies are needed to determine whether this is a clinically useful intervention for peanut allergic patients.

### Epicutaneous immunotherapy (EPIT)

In an effort to optimise allergen administration for food immunotherapy and at the same time reduce the number and severity of immunotherapy-induced side effects, a new route of immunotherapy is currently under investigation. Epicutaneous administration of the allergen avoids highly vascularised sites, which are associated with systemic side effects, but targets professional allergen presenting cells (Langerhans cells of the epidermis) necessary for optimal allergen presentation [33]. A pilot study testing clinical efficacy and safety of epicutaneous immunotherapy in children suffering from cow’s milk allergy showed a tendency towards a higher threshold dose after a 3-month treatment period. Although the results were not statistically significant, the intervention was well tolerated with no observed systemic reactions [34]. A phase I and a phase II trial have recently been initiated for peanut allergy [33].

### Use of peanut vaccine and adjuvants (anti-IgE)

Wood et al. investigated the safety and immunological effects of a vaccine containing modified Ara h1, Ara h2 and Ara h3 (heat/phenol-killed, E.coli-encapsulated, recombinant modified peanut proteins), in 5 healthy volunteers and 10 peanut-allergic adult subjects. The proteins were designed with site-directed mutagenesis to reduce IgE binding, but retain T cell receptor binding. Unfortunately, the administration of the vaccine resulted in frequent allergic reactions (severe reactions in 20%) in the peanut allergic subjects and failed to induce tolerance to the dominant peanut proteins (50% of participants were unable to complete the dosing regimen). The healthy volunteers did not experience any adverse effects, but the vaccine did not prove safe or efficacious for peanut allergy [35].

The use of anti-IgE in peanut allergy was investigated by Leung et al., who conducted a double blind, randomised, dose-ranging trial in 84 peanut allergic patients that received either an anti-IgE molecule (TNX-901) or placebo, once weekly for four weeks. Following treatment, it was shown that a 450 mg dose of anti-IgE increased the threshold of reactivity to peanut from approximately half a peanut at baseline to almost nine peanuts post treatment [36]. The use of anti-IgE has limitations in clinical practice, as it can be expensive as a form of long-term treatment. Currently, it is not known for how long anti-IgE needs to be administered in order to obtain a long-lasting effect of desensitisation to peanut.

A recent pilot study examined the use of anti-IgE (omalizumab) as an adjuvant in peanut oral immunotherapy, with the aim to reduce the number of adverse reactions and minimise in-hospital time and number of visits for participants. 13 peanut allergic children with high peanut specific IgE (median 229 kUA/L) were pre-treated with omalizumab, all of whom tolerated the initial rush desensitisation phase (1st day) with minimal or no rescue therapy. As soon as the maximum maintenance dose was reached (2 g peanut protein, successfully reached by 12/13 subjects), omalizumab was discontinued, but participants continued receiving peanut OIT for a further 12 weeks. A DBPCFC at the end of the 12 weeks showed all subjects tolerating 4 g of peanut protein (8 g peanut flour). During the study, 6 subjects experienced mild or no allergic reactions, 5 subjects had grade 2 reactions (WAO classification) and 2 subjects had grade 3 reactions. It appears that omalizumab may facilitate rapid oral desensitisation in peanut allergic patients with high peanut specific IgE levels at baseline [37].

These interesting findings will require further study with larger trials in order to ascertain the role of anti-IgE in combination with peanut immunotherapy.

### Long-term tolerance versus desensitisation

The potential of food immunotherapy in achieving long-term tolerance (where participants are able to consume the food ad lib without any need for ongoing therapy) versus transient desensitisation (an increase of the threshold of reactivity to the allergen that requires regular therapy in order to be maintained) is still unknown and under investigation.

A randomized controlled trial of egg oral immunotherapy first examined sustained unresponsiveness to egg, following discontinuation of oral immunotherapy for 4–6 weeks (after participants had received maintenance for a total of 22 months). It was shown that 28% of participants were able to maintain their clinical tolerance and were advised to consume the allergen ad lib. All subjects consuming egg ad lib (representing 28% of the group as mentioned above) were able to maintain clinical tolerance after 6–12 months of follow up [23].

For peanut allergy, Blumchen et al. enrolled 23 children aged 3–14 years with confirmed peanut allergy to undergo a rush protocol of OIT for 7 days. The participants subsequently continued with a long-term build-up protocol, where doses were increased every two weeks, up to 500 mg peanut. Once this was achieved, they continued on the same dose for 2 months, before finally stopping OIT completely for two weeks. The investigators repeated the peanut challenge at the end of two weeks with 57% of their subjects maintaining their tolerance, despite having avoided the allergen for 14 days [38].

A recent two-centre US study included 24 children who received peanut OIT for a total of 5 years, and subsequently discontinued oral immunotherapy for 1 month. Participants were subsequently challenged to 5 g of peanut protein and 50% were able to pass this high dose challenge to peanut without reactions. For those who failed the challenge, the eliciting symptom dose (median: 3750 mg) was noted to be much higher than their baseline threshold to peanut [39].

Syed et al. studied 23 participants who underwent peanut OIT for 24 months. Withdrawal of treatment for 3 months resulted in 13 subjects losing their clinical tolerance to peanut. After a further 3 months off therapy, a further 4 participants regained their sensitivity to peanut [40].

Overall, it appears that successful long-term tolerance to peanut after completion of OIT occurs in a small proportion of subjects. The effect is much smaller compared with successful desensitisation, however much larger studies are required to fully address the question of long-term tolerance.

The mechanisms underlying successful immunotherapy and induction of long-term tolerance are still under investigation. However, studies have shown down-regulation of the allergen-specific Th2 response, increase of the Th1 response and induction of regulatory T cells, in association with peanut immunotherapy. In particular, successful peanut immunotherapy has resulted in a peanut-specific IgE decrease, peanut skin prick test decrease, peanut IgG and IgG4 increase, as well as decreased IL-4, IL-5 and IL-13 and increased IL-10 and TGF-b cytokine production [28,31,38,41]. Microarray data has demonstrated down-regulation of genes in several apoptosis pathways in patient T cells, although it is not clear whether these changes included apoptosis of antigen-specific cells as well as total peripheral blood T cells [28]. Clinical immune tolerance has been associated with demethylation of forkhead box protein 3 (FOXP3) CpG sites in antigen-induced regulatory T cells [40]. Generally, most of these immunological changes are similar to those seen in patients receiving immunotherapy for environmental allergens, however more research is needed in the area of food immunotherapy to clearly identify the underlying mechanisms of desensitisation and long-term tolerance.

### Cochrane review on peanut OIT

A Cochrane review, published in 2012, examined the effectiveness and safety of OIT in patients with IgE-mediated peanut allergy. The reviewers identified a small RCT that fit their specified inclusion criteria. They concluded that peanut OIT represents a promising therapeutic approach for the management of peanut allergy. However, the evidence was not sufficient to draw conclusions regarding long-term effectiveness, safety and cost effectiveness of this intervention and it was not recommended for use in clinical practice [42].

It is important to note that since then, many more studies were published on peanut OIT including a large, phase II randomised controlled trial, which included children with peanut allergy of all severities. A more updated Cochrane review is therefore awaited.

## Conclusions

Every new intervention requires a careful assessment of benefits and risks prior to application in clinical practice. So far, published studies on peanut immunotherapy have shown good efficacy in desensitising peanut allergic patients with an acceptable safety profile. Current protocols have used different dosing schedules and varying durations of treatment; patient selection also varied between studies. It is still unclear what the long-term effects of this intervention are and how cessation of treatment will affect individual patients. In addition, OIT protocols are labour-intensive, require dedicated personnel and there are risks involved. The health economics of this novel treatment are also largely unknown.

In summary, peanut immunotherapy presents an exciting, potentially disease-modifying treatment approach for peanut allergy, but is not yet recommended for routine clinical use and should not be attempted outside specialist allergy units.

## Abbreviations

DBPCFC: Double blind placebo controlled food challenge; EPIT: Epicutaneous immunotherapy; OIT: Oral immunotherapy; RCT: Randomised controlled trial; SCIT: Subcutaneous immunotherapy; SLIT: Sublingual immunotherapy.

## Competing interests

KA has no competing interests. AC is a joint patent applicant for a peanut immunotherapy regime.

## Authors’ contributions

The first draft was prepared by Dr Anagnostou. Dr Clark and Dr Anagnostou jointly edited and rewrote subsequent versions. They both reviewed and approved the final manuscript.

## Supplementary Material

Additional file 1 **Key studies of peanut immunotherapy.** Key studies on peanut immunotherapy are described, including the study reference (authors, journal and date of publication), the study design, the route of immunotherapy used and the top dose tolerated orally after immunotherapy treatment, the study protocol (rush, build-up or maintenance), the relevant population (age range in brackets, shown in years), the duration of each study (in days, weeks or months) and the main results (success rate of desensitisation and rate of systemic reactions). Click here for file
