# Supplementary material for: Peanut immunotherapy
Source: Clin Transl Allergy. 2014 Sep 25;4:30. doi: 10.1186/2045-7022-4-30 (PMC4178161; doi:10.1186/2045-7022-4-30)
Supplement: Additional file 1 — Key studies of peanut immunotherapy. Key studies on peanut immunotherapy are described, including the study reference (authors, journal and date of publication), the study design, the route of immunotherapy used and the top dose tolerated orally after immunotherapy treatment, the study protocol (rush, build-up or maintenance), the relevant population (age range in brackets, shown in years), the duration of each study (in days, weeks or months) and the main results (success rate of desensitisation and rate of systemic reactions). [file 2045-7022-4-30-S1.pdf]

**Study reference**	**Study design**	**Route of immunotherapy and top dose tolerated post immunotherapy**	**Study protocol**	**Study population**	**Study duration**	**Main results and rate of systemic reactions during immunotherapy**
Oppenheimer et al, *JACI 1992.*	Randomised Controlled Trial.	Subcutaneous.<br><br>Top oral dose reached post immunotherapy: 8g peanut.	Rush followed by maintenance.	11 subjects with peanut allergy and previous systemic reactions to peanut.<br><br>Mean age: 25 years (14-43).	29 days.	The study was discontinued after 3 active and 1 placebo subjects completed treatment. The 3 active subjects showed a 67-100% decrease in symptoms post immunotherapy.<br><br>Rate of systemic reactions: 13.3%.
Nelson et al, *JACI 1997.*	Randomised controlled trial.	Subcutaneous.<br><br>Top oral dose reached post immunotherapy: 8g peanut.	Rush followed by maintenance.	12 adult subjects with a history of immediate hypersensitivity reactions to peanut.	12 months.	After 1 year of treatment, from the 6 active group subjects, 2/6 tolerated 8g peanut, 1/6 tolerated 4g peanut and 3/6 tolerated less than 2g peanut.<br><br>Rate of systemic reactions: 23% (rush phase)<br>39% (maintenance phase).

				Age range:<br>Active group:<br>18-56 years.<br>Control group:<br>33-46 years.		
Clark et al, *Allergy 2009.*	Open, prospective.	Oral<br><br>Top dose: 2.38g peanut protein.	Build-up followed by maintenance.	4 children with severe peanut allergy.<br><br>Median age: 12.5 years (9-13).	Not stated.	100% successfully desensitised to 2.38g peanut protein post-OIT (compared with 5-50 mg peanut protein pre-OIT).<br><br>Rate of systemic reactions: 0
Jones et al, *JACI 2010.*	Open, prospective	Oral<br><br>Top dose: 3.9g peanut protein.	Rush, build-up and maintenance.	39 children with peanut allergy.<br><br>Median age: 57.5 months (12-111 months).	36 months.	Of 29 subjects who completed the protocol, 93% were successfully desensitised (tolerated 3.9g peanut protein), 7% failed. There was a 25% withdrawal rate.<br><br>Rate of systemic reactions: 15%.
Blumchen et al, *JACI 2010.*	Open, prospective	Oral	Rush, build - up and maintenance.	23 children with peanut allergy.	9 months.	61% successfully desensitised to 0.5-2g peanut<br>17% failed, 22% dropped out.

		Top dose: 4g peanut.		Median age: 5.6 years (3-14).		Rate of systemic reactions: 0
Varshney et al, *JACI 2011.*	Randomised Controlled Trial.	Oral<br><br>Top dose: 5g peanut protein.	Rush, build up and maintenance.	28 children with peanut allergy (active group: 19, placebo group: 9).<br><br>Median age: 6 years (1-16).	12 months.	84% of subjects who completed the study protocol were successfully desensitised to 5g of peanut protein, 16% failed. There was a 32% drop-out rate.<br><br>Rate of systemic reactions: 21%
Anagnostou et al, *CEA 2011.*	Open, prospective.	Oral<br><br>Top dose: 6.6g peanut protein.	Build-up followed by maintenance.	22 children with peanut allergy.<br><br>Median age: 11 years (4-18).	56 weeks	64 % tolerated the top dose (6.6g peanut protein), 22% tolerated a lower dose (800 mg peanut protein) and 9% failed.<br><br>Rate of systemic reactions: 0
Anagnostou et al, *The Lancet 2014.*	Randomised Controlled Crossover Trial.	Oral<br><br>Top dose: 1.4g peanut protein.	Build-up followed by maintenance.	99 children with peanut allergy of any severity.	26 weeks	62% of subjects in the active group that completed the OIT protocol tolerated the top dose of 1.4g peanut protein, 22% tolerated 800mg peanut protein, 16% failed. There was a 10% drop-out rate.

				Median age: 12.4 years (7-16).		Rate of systemic reactions: 1%
Kim et al, *JACI 2011.*	Randomised Controlled Trial.	Sublingual.<br><br>Top dose: 1710 mg peanut protein.	Build up and maintenance.	18 subjects with peanut allergy (active group: 11, placebo group: 7).<br><br>Median age: 5 years (1-11).	12 months	The active group ingested 20 tines more peanut protein than the placebo group (1710g versus 85 mg) post immunotherapy.<br><br>Rate of systemic reactions: 0
Fleischer et al, *JACI 2013.*	Randomised Controlled Trial.	Sublingual.<br><br>Top dose: 496 mg peanut protein.	Build up and maintenance.	40 subjects with peanut allergy.<br><br>Median age: 15 years (12-37).	68 weeks	70% in the active group reached the top dose of 496 mg peanut protein, 30% failed.<br><br>Rate of systemic reactions: 0

Table: Key studies of peanut immunotherapy

Key studies on peanut immunotherapy are described including the study reference (authors, journal and date of publication), the study design, the route of immunotherapy used and the top dose tolerated orally after immunotherapy treatment, the study protocol (rush, build-up or maintenance), the relevant population (age range in brackets, shown in years), the duration of each study (in days, weeks or months) and the main results (success rate of desensitisation and rate of systemic reactions).
